# Differential effect of lactate in predicting mortality in septic patients with or without disseminated intravascular coagulation: a multicenter, retrospective, observational study

**DOI:** 10.1186/s40560-019-0389-x

**Published:** 2019-06-24

**Authors:** Daisuke Hasegawa, Kazuki Nishida, Yoshitaka Hara, Takahiro Kawaji, Kazuhiro Moriyama, Yasuyo Shimomura, Daisuke Niimi, Hidefumi Komura, Osamu Nishida

**Affiliations:** 1 Department of Anesthesiology and Critical Care Medicine, School of Medicine, Fujita Health University, 1-98 Dengakugakubo, Kutsukake-cho, Toyoake, Aichi 470-1192 Japan; 2 Department of Biostatistics, Graduate School of Medicine, Nagoya University, Nagoya, Aichi Japan; 3 Laboratory for Immune Response and Regulatory Medicine, School of Medicine, Fujita Health University, Toyoake, Aichi Japan; 4 Department of Anesthesiology, Nishichita General Hospital, Tokai, Aichi Japan

**Keywords:** Sepsis, Disseminated intravascular coagulation, Lactate, Mortality

## Abstract

**Background:**

We examined whether high lactate level in septic patients was associated with 90-day mortality based on the patients’ disseminated intravascular coagulation (DIC) status.

**Methods:**

We conducted a multicenter, retrospective, observational study of patients admitted to the intensive care unit (ICU) with a suspicion of severe infection and diagnosed with sepsis. Regression analyses were performed to estimate the interaction effect between DIC status and the lactate level. Then, the association between the lactate level and 90-day mortality was assessed in the DIC and non-DIC subgroups.

**Results:**

The data of 415 patients were analyzed. We found a significant interaction between DIC status and the lactate level for predicting 90-day mortality (*p*_interaction_ = 0.04). Therefore, we performed a subgroup analysis and found that high lactate concentration was significantly associated with 90-day mortality in the DIC group (odds ratio = 2.31, *p* = 0.039) but not in the non-DIC group.

**Conclusions:**

In patients with DIC, a high lactate level significantly predicted 90-day mortality; no such association was found in the non-DIC group. Thus, DIC status may serve as a possible effect modifier of lactate level in predicting mortality in patients with sepsis.

## Background

Sepsis is among the most common causes of death among hospitalized patients [1], and its incidence has been increasing annually [2]. However, remarkable advances in the management of sepsis have been made in recent years, leading to significant improvements in survival [3]. As the recent Sepsis-3 definition includes the presence of organ dysfunction as a diagnostic criterion [4], increased attention has been paid to the management of organ dysfunction.

Organ dysfunction can be identified as an acute change of ≥ 2 points in the total Sequential (sepsis-related) Organ Failure Assessment (SOFA) score [5] resulting from infection. Although the prevention and management of infection-induced organ dysfunction is a key to the treatment of septic patients, the pathophysiological mechanisms underlying organ dysfunction in patients with sepsis are not entirely known. Among the causes of organ dysfunction in sepsis, disseminated intravascular coagulation (DIC) is considered to be partly involved [6]. The Japanese Association for Acute Medicine—disseminated intravascular coagulation (JAAM-DIC) diagnostic criteria consist of the prothrombin time ratio, systemic inflammatory response syndrome (SIRS) score, fibrin degradation product, and the count and/or reduction rate of platelets [7]. This scoring system was created for diagnosing septic DIC and was shown to have a good prognostic value for predicting mortality in patients with sepsis [8]. The defining characteristic of this score is that it reflects the intensity of inflammation and severity of coagulopathy based on the concept of crosstalk between coagulation and inflammation [9].

Although lactate is known to be an anaerobic metabolite and is considered to be a good predictor of sepsis severity, its elevation can occur for various other causes aside from sepsis. However, lactate level elevation coupled with severe inflammation and coagulopathy may reflect the early phase of organ dysfunction as microcirculatory dysfunction caused by persistent microthrombosis can induce a reduction in blood flow to the tissues and cause organ dysfunction in a later phase. Therefore, we hypothesized that using the lactate value in predicting mortality varies according to coagulation condition. In this study, we evaluated the usefulness of the lactate level in predicting mortality among patients with sepsis, based on the presence or absence of DIC.

## Methods

This retrospective observational study was conducted at a Japanese university hospital and a Japanese community hospital (Fujita Health University Hospital and Nishichita General Hospital, respectively). A total of 415 patients aged ≥ 18 years who were admitted to the intensive care unit (ICU) with the suspicion of severe infection and diagnosed with sepsis with the use of the Sepsis-3 definition between January 2013 and December 2017 were selected for this study (between May 2015 and December 2017 in Nishichita General Hospital since the ICU in the hospital was established in May 2015). Our primary endpoint was 90-day mortality, and our secondary endpoint was 28-day mortality. Based on previous literature, we defined a suspicion of severe infection based on a combination of culture sampling and the initiation of antibiotic treatment. If the antibiotic was given prior to the culture, the culture sample must have been obtained within 24 h. If the culture sampling occurred first, the antibiotic must have been ordered within 72 h [10]. The exclusion criterion was unknown outcomes (alive or dead within 90 days).

We collected data on the patients’ medical history, physical examination results, and laboratory results from a retrospective review of medical records. Table 1 shows the classification according to the JAAM-DIC scoring system [7].

**Table 1:** Japanese Association for Acute Medicine—disseminated intravascular coagulation scoring system

	Score
SIRS score	
≥ 3	1
0–2	0
Platelet count	
< 80 × 10^9^/L or > 50% decrease within 24 h	3
≥ 80 < 120 × 10^9^/L or > 30% decrease within 24 h	1
≥ 120 × 10^9^/L	0
Prothrombin time (value of patient/normal value)	
≥ 1.2	1
< 1.2	0
Fibrin/fibrinogen degradation products	
≥ 25 mg/L	3
≥10 < 25 mg/L	1
< 10 mg/L	0
Diagnosis	
Disseminated intravascular coagulation	≥ 4

*SIRS* systemic inflammatory response syndrome

SIRS was defined according to the American College of Chest Physicians/Society of Critical Care Medicine [11] criteria. Scoring ≥ 4 points on the JAAM-DIC scale was the qualifying criterion for a diagnosis of DIC.

Lactate levels were measured at the time of admission to the intensive care unit, and we chose a cutoff value of 4 mmol/L for lactate because a lactate value equal or more than 4 mmol/L was demonstrated to be an independent risk factor for mortality in sepsis [12]. We classified patients with a lactate value ≥ 4 mmol/L as “high lactate.” Similarly, we classified patients with a lactate value < 4 mmol/L as “low lactate.”

Our hypothesis was to examine whether the effect of lactate on 90-day mortality depends on DIC/non-DIC status. In this study, this corresponds to the interaction effect between lactate and DIC status. Thus, we assessed for an interaction effect in the multivariable logistic regression model adjusted for age, sex, and SOFA score. To examine the actual relationship between lactate and 90-day mortality, additional subgroup analysis stratified by DIC status is required [13]. In addition, we conducted Cox proportional hazard regression analysis to confirm the main effect of lactate performance on mortality in each DIC/non-DIC group, with survival up to 90 days as the time variable. Covariates included age, sex, and SOFA score, which were identical to those included in the multiple logistic regression model [13]. The numerical values in the text and tables represent the median (interquartile range) unless otherwise noted. A *p* value < 0.05 was considered statistically significant. All statistical analyses were performed using EZR (version 1.31; Saitama Medical Center, Jichi Medical University, Saitama, Japan), which is a graphical user interface for R (version 3.2.2; R Foundation for Statistical Computing, Vienna, Austria) [14].

Due to the anonymous nature of the data, the requirement for informed consent was waived. The study protocol was approved by the institutional review board of Fujita Health University and Nishichita General Hospital (approval no. HM18–190 and 30–25, respectively).

## Results

**Fig. 1:**
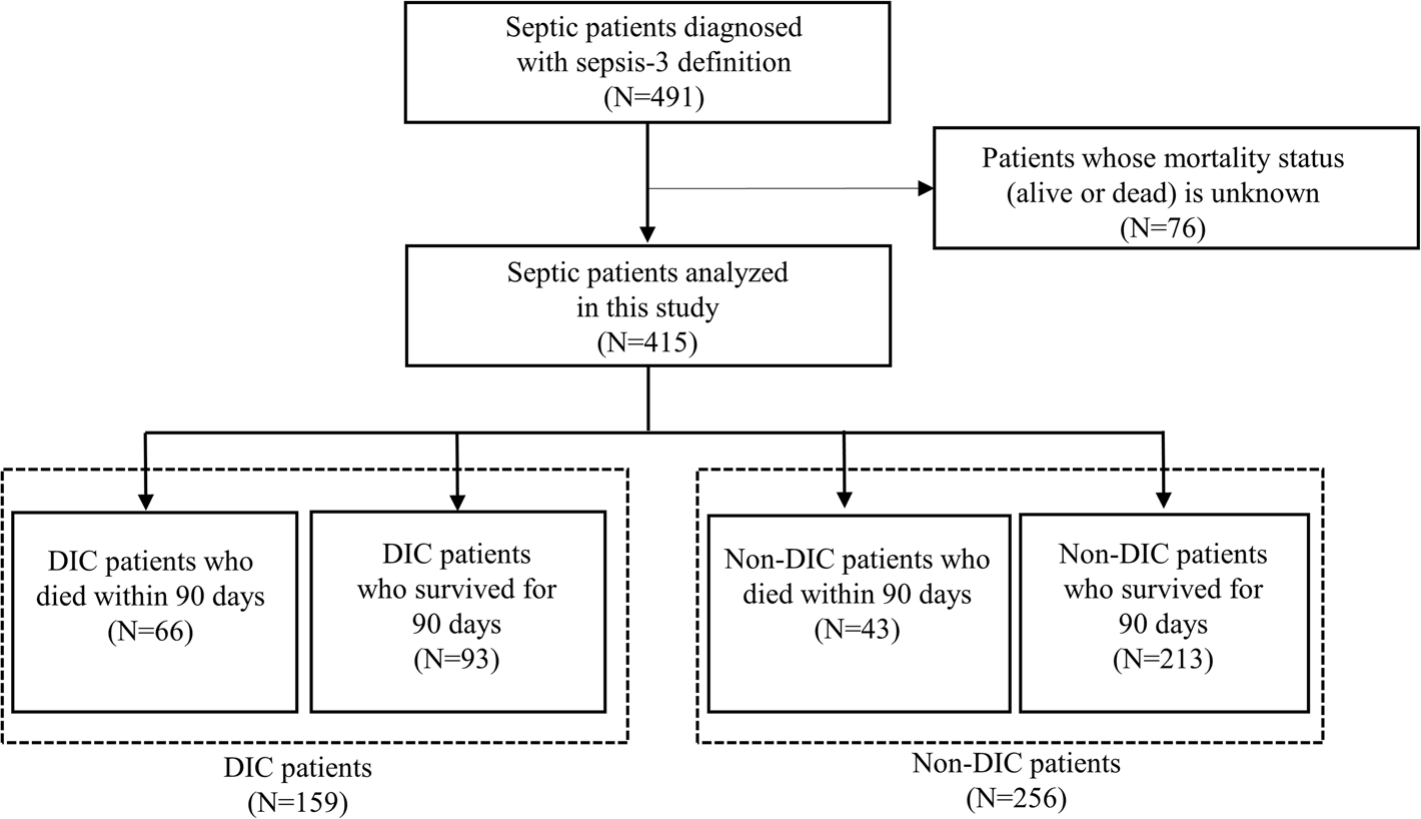
Patient flow diagram of this study. DIC, disseminated intravascular coagulation; *N*, number

On the basis of the exclusion criteria, 76 patients were excluded (57 patients from Fujita Health University Hospital and 19 from Nishichita General Hospital), and finally, 415 patients were included in this study (346 patients from Fujita Health University Hospital and 69 from Nishichita General Hospital). Figure 1 shows a flow diagram of this research. Table 2 displays the baseline characteristics of all patients included in this study by DIC status.

**Table 2:** Baseline characteristics of all patients included in this study by DIC status (*N*=415)

Parameter	All (*n* = 415)	DIC group (*n* = 159)	Non-DIC group (*n* = 256)	*p* value
Men, *n*/%	281/68%	111/70%	170/66%	0.518
Age, years	72 (63–78)	71 (64–79)	72 (62–77)	0.6715
BMI	21 (18.5–23.6)	21 (18.4–23.7)	21 (18.6–23.5)	0.9842
SOFA score	7 (4–11)	10 (7–13)	6 (3–9)	< 0.001
SIRS score	2 (2–3)	3 (2–3)	2 (1–3)	< 0.001
APACHE II score	23 (17–30)	26 (20–35)	21 (16–28)	< 0.001
Lactate value, mmol/L	1.7 (1.1–3.3)	2.6 (1.3–4.6)	1.6 (1.0–2.6)	< 0.001
Source of sepsis				
Abdominal, *n*/%	158/38%	59/37%	99/39%	0.756
Respiratory, *n*/%	134/32%	49/31%	85/33%	0.666
Intravascular, *n*/%	45/11%	18/11%	27/11%	0.871
Skin/joint, *n*/%	42/10%	8/5.0%	34/13%	0.007
Urinary tract, *n*/%	18/4.3%	13/8.2%	5/2.0%	0.005
Central nervous system, *n*/%	4/0.97%	1/0.62%	3/1.2%	> 0.999
Unknown, *n*/%	14/3.4%	11/6.9%	3/1.2%	0.003
ICU stay, days	7 (4.0–13)	9 (5.0–18)	6 (3.8–11)	< 0.001
Hospital stay, days	49 (24–99)	50 (26–93)	49 (23–101)	0.658
28-day mortality, *n*/%	60/14.5%	35/22.0%	25/9.8%	< 0.001
90-day mortality, *n*/%	109/26.3%	66/41.5%	43/16.8%	< 0.001

Data are presented as median and interquartile ranges (25–75% percentile) or as absolute frequencies with percentages. *APACHE* Acute Physiology and Chronic Health Evaluation, *BMI* body mass index, *JAAM-DIC* Japanese Association for Acute Medicine—disseminated intravascular coagulation, *ICU stay* intensive care unit stay, *SIRS* systemic inflammatory response syndrome, *SOFA* Sequential (sepsis-related) Organ Failure Assessment

The subjects included 281 men and 134 women with a median age of 72 (range, 62.5–78) years. Their median SOFA score was 7.0 (4.0–11); the median Acute Physiology and Chronic Health Evaluation (APACHE) II score [15] was high, at 23 (17–30). The patients’ median lactate level was also high, at 1.7 (1.1–3.3) mmol/L. The 28-day and 90-day mortality rates were 14.5% and 26.3%, respectively.

Tables 3 and 4 show the adjusted odds ratios (ORs) for DIC defined by JAAM-DIC score and high lactate value in predicting 28- and 90-day mortality, respectively.

**Table 3:** Odds ratios of the DIC and high lactate value in predicting 28-day mortality

Parameter	Odds ratio	95% CI	*p* value
DIC	1.72	0.87–3.39	0.121
High lactate level	0.73	0.2–2.67	0.635
Age	1.04	1.01–1.06	0.009
Male	1.65	0.85–3.2	0.137
SOFA score	1.08	1–1.16	0.062
DIC and high lactate	2.06	0.44–9.57	0.355

The model was adjusted for age, sex, and SOFA score

DIC: JAAM-DIC score, Japanese Association for Acute Medicine—disseminated intravascular coagulation score, ≥ 4. High lactate level: lactate value ≥ 4 mmol/L

*CI* confidence interval, *SOFA* Sequential (sepsis-related) Organ Failure Assessment

**Table 4:** Odds ratio of the DIC and lactate value in predicting 90-day mortality

Parameter	Odds ratio	95% CI	*p* value
DIC	2.2	1.27–3.8	0.005
High lactate level	0.54	0.18–1.68	0.289
Age	1.03	1.01–1.05	0.005
Male	1.53	0.9–2.59	0.113
SOFA score	1.06	1.0–1.13	0.067
DIC and high lactate	4.13	1.07–15.97	0.04

The model was adjusted for age, sex, and SOFA score

DIC: JAAM-DIC score, Japanese Association for Acute Medicine—disseminated intravascular coagulation score, ≥ 4. High lactate level: lactate value ≥ 4 mmol/L. *CI* confidence interval, *SOFA* Sequential (sepsis-related) Organ Failure Assessment

After adjusting for age, sex, and SOFA score, the DIC defined by JAAM-DIC score ≥ 4 was significantly associated with 90-day mortality (OR = 2.2, 95% CI 1.27–3.8, *p* = 0.005). By contrast, a high lactate value was not significantly associated with either 28- or 90-day mortality (OR = 0.73, 95% CI 0.2–2.67, *p* = 0.635 and OR = 0.54, 95% CI 0.18–1.68, *p* = 0.289, respectively). The results further revealed a statistically significant interaction between the DIC status and lactate value for predicting 90-day mortality (*p*_interaction_ = 0.04) (Table 4).

Therefore, we performed subgroup analyses classified by the presence or absence of DIC. Tables 5 and 6 display the adjusted ORs for the lactate value as a predictor of 28- and 90-day mortality, respectively, in the DIC group using the multivariable logistic regression model.

**Table 5:** Odds ratio of the lactate value in predicting 28-day mortality in the DIC group

Parameter	Odds ratio	95% CI	*p* value
High lactate level	1.47	0.6–3.59	0.4
Age	1.02	0.99–1.05	0.193
Male	1.3	0.54–3.13	0.555
SOFA score	1.1	0.99–1.22	0.071

The model was adjusted for age, sex, and SOFA score

High lactate level: lactate value ≥ 4 mmol/L

*CI* confidence interval, *DIC* disseminated intravascular coagulation, *SOFA* Sequential (sepsis-related) Organ Failure Assessment

**Table 6:** Odds ratio of the lactate value in predicting 90-day mortality in the DIC group

Parameter	Odds ratio	95% CI	*p* value
High lactate level	2.31	1.04–5.13	0.039
Age	1.02	0.99–1.04	0.195
Male	1.09	0.53–2.26	0.81
SOFA score	1.07	0.98–1.17	0.143

The model was adjusted for age, sex, and SOFA score

High lactate level: lactate value ≥ 4 mmol/L

*CI* confidence interval, *DIC* disseminated intravascular coagulation, *SOFA* Sequential (sepsis-related) Organ Failure Assessment

Although high lactate level was not significantly associated with 28-day mortality (OR = 1.47, 95% CI 0.6–3.59, *p* = 0.4), it was significantly associated with 90-day mortality in the DIC group (OR = 2.31, 95% CI 1.04–5.13, *p* = 0.039).

Tables 7 and 8 show the adjusted ORs for a high lactate value in predicting 28- and 90-day mortality, respectively, in the non-DIC group using the multivariable logistic regression model.

**Table 7:** Odds ratio of the lactate value in predicting 28-day mortality in the non-DIC group

Parameter	Odds ratio	95% CI	*p* value
High lactate level	0.67	0.18–2.5	0.555
Age	1.06	1.01–1.1	0.015
Male	2.16	0.76–6.1	0.146
SOFA score	1.04	0.92–1.17	0.515

The model was adjusted for age, sex, and SOFA score

High lactate level: lactate value ≥ 4 mmol/L

*CI* confidence interval, *DIC* disseminated intravascular coagulation, *SOFA* Sequential (sepsis-related) Organ Failure Assessment

**Table 8:** Odds ratio of the lactate value in predicting 90-day mortality in the non-DIC group

Parameter	Odds ratio	95% CI	*P* value
High lactate level	0.49	0.16–1.54	0.223
Age	1.04	1.01–1.08	0.009
Male	2.15	0.96–4.82	0.062
SOFA score	1.05	0.96–1.16	0.301

The model was adjusted for age, sex, and SOFA score

High lactate level: lactate value ≥ 4 mmol/L

*CI* confidence interval, *DIC* disseminated intravascular coagulation, *SOFA* Sequential (sepsis-related) Organ Failure Assessment

Similar to the DIC group, a high lactate value was not significantly associated with 28-day mortality in the non-DIC group (OR = 0.67, 95% CI 0.18–2.5, *p* = 0.555). It was also not significantly associated with 90-day mortality in the non-DIC group (OR = 0.49, 95% CI 0.16–1.54, *p* = 0.223). As for the results of the Cox proportional hazard regression analysis with 90-day follow-up periods, the hazard ratio for lactate in the DIC group was 1.82 (95% CI 1.07–3.10, *p* = 0.028), whereas the hazard ratio for lactate in the non-DIC group was 0.58 (95% CI 0.20–1.62, *p* = 0.296).

## Discussion

We examined whether changes in lactate level were useful for predicting mortality based on the patient’s DIC status, as defined by the JAAM-DIC diagnostic criteria. We found that a high lactate value was associated with 90-day mortality in the DIC group only.

Although we did not find a statistically significant relationship between lactate level and 90-day mortality, which some previous studies have found, we detected a difference in the association between lactate and 90-day mortality between the DIC and non-DIC groups. Since previous studies did not clarify the proportion of septic DIC patients based on Sepsis-3 and JAAM-DIC diagnostic criteria, it may be possible that previous studies which found an association between lactate and predicted mortality included a higher proportion of septic DIC patients compared to studies which did not detect lactate’s predictive value of mortality. To the best of our knowledge, this is the first reported study to have investigated the predictive value of lactate for mortality based on DIC status in septic patients. Previous studies have focused on a single parameter; thus, a strength of our investigation is that we examined the ability of lactate level to predict mortality based on stratification by DIC status.

Although the mechanisms underlying organ dysfunction in patients with sepsis remain poorly understood, DIC is thought to be partly involved in organ dysfunction pathogenesis in sepsis [6]. The Sepsis-3 guidelines define sepsis as life-threatening organ dysfunction caused by a dysregulated host response to infection [4]. Recently, researchers investigated the possible molecular biologic mechanisms of organ dysfunction caused by a dysregulated host response to infection. As one of the innate immune response systems, neutrophil extracellular traps (NETs) [16] released by neutrophils seem to play a role in early host defense against bacterial dissemination. NETs capture bacteria by forming immunothrombosis in local areas [17]. However, uncontrolled, excessive immunothrombosis involving severe inflammation may lead to DIC and possibly cause organ dysfunction by preventing adequate blood supply to the tissues [18]. In addition, activated platelets induce NETs to ensnare bacteria in septic blood [19]. It has also been shown that platelets and NETs cooperate to form blood clots in vitro [20]. The JAAM-DIC score includes all concepts related to a dysregulated host response that can be caused by excessive immunothrombosis and subsequent microcirculatory disorder which, theoretically, could lead to organ dysfunction.

Lactate values can be elevated for various reasons in sepsis. In addition to hypoxemia, mitochondrial insufficiency in metabolizing pyruvate, caused by excessive stress, can cause an elevation of lactate levels in septic patients. However, by combining the lactate value with the JAAM-DIC score, patients with poor prognoses and potential subsequent organ dysfunction could be detected at an earlier phase. This could explain why hyperlactaemia was associated with mortality in the DIC but not in the non-DIC group. We believe that our findings can help elucidate the pathophysiology underlying organ dysfunction induced by sepsis.

The medication ART123 [21], or recombinant human thrombomodulin alpha, targets sepsis-induced coagulopathy; it has gone through a phase 2b study [22] and is now being investigated in a randomized, double-blinded, placebo-controlled, phase 3 study. Theoretically, this medication has the most impact on patients with microcirculatory dysfunction and subsequent organ dysfunction caused by immunothrombosis. Thus, the findings of our study may aid in identifying subgroups of patients that can benefit from this medication by stratifying them by lactate values and the JAAM-DIC score.

In this study, we did not observe any significant differences between the DIC and non-DIC groups when examining the association between lactate level and 28-day mortality. We assume that this could be explained by the unique Japanese critical care system. In Japan, most medical costs are covered by public health insurance, and treatment is seldom withdrawn for patients with dismal prognoses. Thus, it is possible that severely ill patients were still alive at 28 days but not at 90 days. In this sense, we believe that 90-day mortality is a more useful outcome in this study than 28-day mortality; thus, we chose 90-day mortality as the primary outcome.

This study is not without limitations. First, because this was a retrospective study with a limited sample size, the risk of residual confounding and the risk of type I error remain. Additional work is necessary to provide more definitive data. Second, all the patients analyzed in this study were Asians; therefore, it is unclear whether our findings apply to other ethnic groups. However, this was a preliminary study to identify possible associations. Multicenter prospective studies are warranted to confirm the findings of this study.

## Conclusions

In septic patients with DIC, elevated lactate levels significantly predicted 90-day mortality; in contrast, the lactate value did not predict 28- or 90-day mortality in patients within the non-DIC group. Further studies are warranted to investigate the effect of lactate on mortality in patients with sepsis.

## Data Availability

The datasets analyzed during the current study are available from the corresponding author on reasonable request.

## Abbreviations

APACHE: Acute Physiology and Chronic Health Evaluation
CI: Confidence interval
DIC: Disseminated intravascular coagulation
ICU: Intensive care unit
JAAM-DIC: Japanese Association for Acute Medicine—disseminated intravascular coagulation
NET: Neutrophil extracellular trap
OR: Odds ratio
SIRS: Systemic inflammatory response syndrome
SOFA: Sequential (sepsis-related) Organ Failure Assessment
